# Comprehensive Assessment of Ameliorative Effects of AMF in Alleviating Abiotic Stress in Tomato Plants

**DOI:** 10.3390/jof7040303

**Published:** 2021-04-15

**Authors:** Murugesan Chandrasekaran, T. Boopathi, Paramasivan Manivannan

**Affiliations:** 1 Department of Food Science and Biotechnology, Sejong University, Seoul 05006, Korea; 2 Department of Biology, Gandhigram Rural Institute, Tamilnadu 624302, India; iamboopathy@gmail.com; 3 Department of Microbiology, Bharathidasan University, Tamilnadu 620024, India; manivannan3065@gmail.com

**Keywords:** arbuscular mycorrhizal, salt stress, drought stress, heavy metal stress, temperature stress

## Abstract

Population growth and food necessity envisaged the dire need for supplementation to a larger community balance in food production. With the advent of the green revolution, agriculture witnessed the insurrection of horticultural fruit crops and field crops in enormous modes. Nevertheless, chemical fertilizer usage foresees soil pollution and fertility loss. Utilization of biocontrol agents and plant growth promotion by microbial colonization enrooted significant restoration benefits. Constant reliability for healthy foods has been emancipated across the globe stressing high nutritive contents among indigenous field crops like tomato (*Solanum lycopersicum*). However, stress tolerance mechanisms and efficient abatement require deeper insights. The applicability of arbuscular mycorrhizal fungi (AMF) poses as an ultimate strategy to minimize the deleterious consequences of abiotic stress such as salt, drought, temperature and heavy metal stress sustainably. The rational modality employing the application of AMF is one of significant efforts to lessen cell damages under abiotic stress. The novelty of the compilation can be redressed to cohesive literature for combating stress. The literature review will provide agricultural scientists worldwide in providing a rational approach that can have possible implications in not only tomato but also other vegetable crops.

## 1. Introduction

Tomato (*Solanum lycopersicum*) remains the most widely cultivated and largely consumed vegetables, next to potato universally. Tomato is regarded as a staple food crop to all sections of the economy irrelevant of developed or underdeveloped nations over a wide array of population. Large area under cultivation of nearly 4.2 million ha yielding 100 million tons per year enunciates the ultimate significance for the vegetable crop. Horticultural applicability of the fleshy vegetable nature of tomato has invigorated for its wide applications in food and feed ingredients. Major incorporations in salads and all varieties of dishes ranging from pasta, pizza, and other bakery products render them as an effective food supplement. Health-enhancing components in tomato comprise vitamins like C and E, beta-carotene, lycopene, flavonoids, and lutein. Macronutrient contents like potassium (K), phosphorous (P), magnesium (Mg), and calcium (Ca) along with trace elements composition of iron (Fe), manganese (Mn), zinc (Zn), and copper (Cu) stress the biocompatibility of staple and sustainable nature of tomato [1]. When foods containing tomato are subjugated for nutritional capabilities in arresting debilitating diseases like cancer and cardiovascular complications provide explicit strategies for nutraceutical benefits addressing food as medicine [2,3,4].

Tomato production and yield despite of variability patterns of region-specificity, soil fertility, and productivity are hampered by stress complications like drought, salt stress, temperature and environmental complexities especially heavy metal contamination accounting for nearly 70% loss eventually affecting food security. Subsequent stress emancipated through abiotic stress modalities addresses a large-scale hurdle in tomato plants growth and productivity throughout the world necessitating an effective alternate mode in efficient abatement of plant stress.

**Figure 1:**
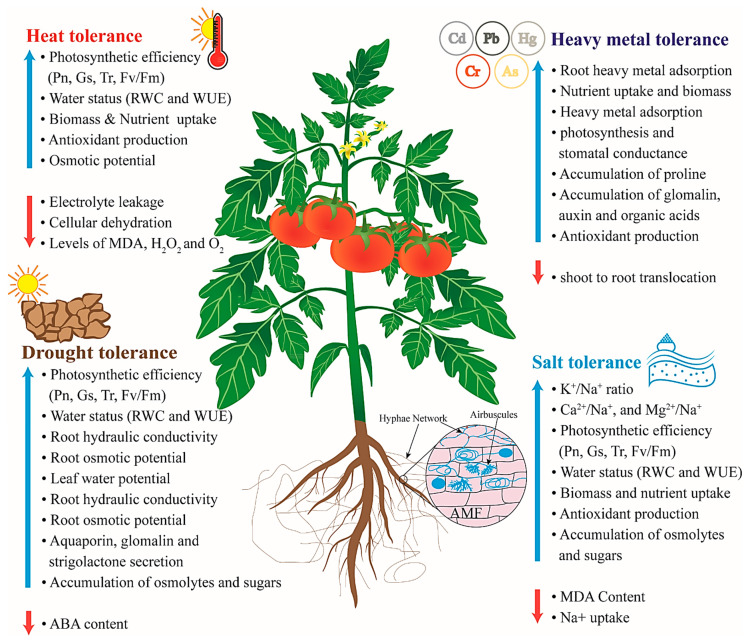
Effects of arbuscular mycorrhizal inoculation on abiotic stress alleviation in tomato.

Precisely, abiotic stress factors are on an alarming rise emphasizing the requirement for arresting the devastating predicaments of salt stress, drought and soil contamination that accounts for vulnerability in sustainable agriculture of over 10% of arable land ensuing major yield loss of nearly 50% inclusive of key horticultural crops like tomato. Therefore, improvement of tolerance of plants against abiotic stresses remains a major focus of agricultural research. In day-to-day agriculture, the sustainability of crop productivity and yield are largely affected by abiotic stress thus corroborating to greater hazards both for the soils and the plant immunity. Climate change and ever-increasing industrialization are attributed to high range of abiotic stresses that affect yield and productivity. Thus, cost-effective strategies for the application of arbuscular mycorrhizal fungi (AMF) were assessed for biofertilization, commercialization, and commodity selection for ensuring maximal benefits. Many studies have demonstrated an ameliorative effect of AMF in alleviating abiotic stress in tomato plants (Figure 1). Holistically, there have been ample reviews about the AMF application for improvising tomato cultivation; the present review combines multi-disciplinary approaches in employing AMF and the potential benefits. Innovativeness can be confronted on the compilation of updated AMF profiles and their mechanistic molecular implications of AMF in abiotic stress amelioration for enhanced tomato productivity. Tolerating heat is attributed to many factors as enlisted in Figure 1. Heat stress poses serious consequences due to global warming. Reduction in electrolyte leakage, malondialdehyde (MDA), H_2_O_2_ and O_2_^−^ levels along with escalating photosystem II (PS II), chlorophyll concentration, photosynthetic rate, tomato seedling growth rate, antioxidant levels, nutrient uptake, and biomass are discussed appropriately in the review. Similarly, the factors for drought tolerance (increased biomass, nutrient uptake, photosynthetic rate (Pn), stomatal conductance (Gs), water uptake, root hydraulic conductivity, root osmotic potentials, leaf water potentials, water use efficiency (WUE), stress hormone levels, and osmolytes accumulation), heavy metal tolerance (increase in root changes, nutritional status, heavy metal adsorption, secondary metabolites, glomalin, auxin, organic acids, antioxidants and nutrient uptake), and salt tolerance (increase in biomass, nutrient uptake, K^+^/Na^+^ ratio, Ca^2+^/Na^+^, Mg^2+^/Na^+^, Pn, Gs, transpiration rate (Tr), water uptake, antioxidant status, osmolyte accumulation and diminished MDA content) that require deeper and intricate mechanism revelation are concisely analyzed.

## 2. Arbuscular Mycorrhizal Fungi

AMF, belonging to the Glomeromycota, are obligate biotrophs that establish mutualistic symbiotic associations, with most flowering plants revealing plant beneficiary attributes for a wide range of plants [5,6]. AMF principally are segregated into four classes inclusive under Glomerales, Archaeosporales, Paraglomerales, and Diversisporales, which are further sub-divided based on morphological parameters into 25 genera grouped under the sub-phylum [7]. Upon successful colonization, the fungi occupy a vast surface area involving extraradical hyphae networking like cloud embedded in a matrix of the root cortex. These hyphae upon increased accumulation render the soil mass enhancement and aid in augmented quenching of nutrient uptake stressing soil fertility and alternatively for plant growth promotion [8]. Nutrient exchange between the partners reveals the involvement of specialized structures termed arbuscules in the fungi that assist in nutrient exchange and efficient uptake. The mycorrhizal pathway renders the effective uptake of P, N and micronutrients that are not easily available for plant uptake from the soil through thick external mycelial mass surpassing the depletion zone enveloping the plant roots. In return, the plants’ aid compatible, mutual benefits to the AMF in an obligate biotrophic association by providing carbohydrates as a food source [9,10,11], and life cycle management through lipids utilization [12]. AMF not only facilitates nutrient uptake but also plant resistance mechanisms against various stress mechanisms like salinity, drought, and temperature fluctuations together with heavy metal toxicity [8]. AMF also acts as an essential, facultative, obligate endosymbionts offering sustainable agriculture by ensuring ecosystem modulation and phytobiome engineering for successful crop production [13].

## 3. AMF-Mediated Growth and Yield Enhancement in Tomato

Soil fertility throughout the world depends on several soil parameters focusing plant growth nutrient availability that confronts to an increased usage of chemical fertilizers to capitalize huge benefits based on production and yield [14,15]. Many researchers and agriculturalists worldwide have proved AMF as an efficient growth regulator irrespective of nutrient availability ranging from poor to medium fertility. Further, AMF inoculation and optimistic response on tomato yield had adhered to critical effects in combinatorial benefits with (nitrogen phosphorus potassium) NPK fertilizers or varying P levels effectuating plant growth through enhancement of nutrient availability [16,17]. Even under low P availability, particular AMF for a definite crop provide the biofertilizer appropriateness for novel and innovative eco-sustainable practice for increased crop profitability for growers. These facts represent AMF inoculation under low P availability for enhanced P uptake, enriching biomass and thereby establishing high plant growth [18,19] and tissue level escalated deposition of P content [20].

*Funneliformis mosseae* altogether resulted in augmented total yield and slender rise in fruit size show pertinent effects of AMF on tomato production [21]. Application of commercial AMF inoculum (*F. mosseae* and *Septoglomus constrictum*) caused an increase in the overall height of tomato plants despite chemical fertilization in inoculated plants (48.4 cm plant^−1^) than non-inoculated plants (39.7 cm plant^−1^) emphasizing 18% gain. When the effect of mycorrhizal inoculation was studied for overall yield impact, 23% gain was found in inoculated plants (57.1 t ha^−1^) compared to non-inoculated (43.93 t ha^−1^) counterparts. Quantitative assessments of fruit numbers yield were escalated to 35 numbers in inoculated plants (30.6 fruit plant^−1^) rather than non-inoculated ones (19.9 fruit plant^−1^). The above results affirm that AMF inoculation resulted in fruit numbers regardless of the fertilization criterion in tomato. Hence, AMF that are newly introduced than the indigenous AMF unlike the conventional practices employing *Trichoderma* that reveal the contrary of inoculation benefits compared to AMF. Nevertheless, these results were derived from rigorous field trials indicating the need for further research in authenticating long-term field applications [21]. AMF inoculation had also positive impacts upon flowering that have inducing effects on overall yield consequently [17]. *Rhizophagus intraradices* inoculation articulated elevated P revival better than the plants which were not inoculated [17]. *Rhizophagus etunicatum* under enriched P environment widened leaf surface area before flowering and total flower production per plant [16]. The results summarily show an increased flower and fruit production upon AMF inoculation in both the cultivars and escalated seed numbers per fruit in one cultivar. Thus, the impact of mycorrhizal infection upon plant reproduction, and vegetative growth will increase the significance of mycorrhizal research in agriculture, forestry, and land reclamation ensuring food production and security for meeting the issue of global population explosion.

## 4. Drought Stress

Among the abiotic stresses, drought stress accounts for water unavailability posing as the sole criteria in deciding the growth and development of tomato crop plants eventually causing reduction in nutrient uptake hampering the efficiency of production and yield [22]. The molecular mechanism of the stress-induced deficit can be attributed to obstacles in selective permeability which alter active transport characterized phenomenally to the reduction of transpiration rates [22], which advertently result in the culmination of physiology and metabolic pathways hampering that ultimately leads to lessened plant growth due to plant growth promotion, respiration vs. photosynthesis and hindered translocation of photo-assimilates. The effective way to combat water deficit stress has been focused on AMF symbiosis apart from agricultural methodologies and the ecosystem perspective for sustainable agricultural practices [23,24].

The mechanism of AMF symbiosis in mitigating water deficit corresponds to a combinatorial benefit emancipated by nutritional, physical, and cellular effects resulting in the primary effect [25]. The mechanisms that underlie the above said phenomenon are correlated to, (1) higher nutrients absorption [26], (2) articulation of water uptake by external hyphal mass by escalating hydraulic conductivity rendering high water status for the host plants [26], (3) osmotic adjustment [27], (4) increase in antioxidant activity [28], (5) modification of hormonal balance [29]. Non-availability of water in surplus amounts has key retardations ranging from gene expression, secondary metabolite synthesis which in turn affects yield and growth parameters on the tomato plants [30,31,32,33]. There are plentiful reports that establish the AMF symbiosis with plants in resisting drought havoc [34,35,36,37,38]. The variability patterns in the data can be assorted to the ubiquity of the host plants and non-correlation of the AMF under various drought stresses based on region-specific anomalies [39].

### 4.1. Plant Growth and Nutrient Uptake

AM symbiosis and colonization has been amply reported to enhance plant growth and productivity despite drought stress and water deficit conditions [25]. Tomato seedlings inoculated with *R. etunicatum* produced higher dry biomass than non-mycorrhizal plants [40]. *R. clarum* encouraged higher growth in colonized tomato plants aerial biomass than in root biomass under drought stress [34] because AM colonization causes a proportionally greater allocation of carbohydrates to the shoot than to the root tissues [41]. AMF colonization also provides an increase in leaf surface area of inoculated plants and shoot biomass, wherein wild type tomato revealed a high rate of mycorrhizal population than mutant tomato plants under drought stress [42]. *S. constrictum* inoculation under water stress overpowered *S. deserticola* pretreatment indicating mycorrhizal colonization to have profound effects with 14–18% high root and shoot dry weight rather than the non-colonized plants [28]. Moreover, *F. mosseae*, *R. irregulare,* and *R. etunicatum* colonization showed similar results and thereby characterizing growth promotion as a positive regulator with the degree of stress mediated by collective AMF-inoculation [43]. The increase in root biomass eventually can be correlated as crucial since increased soil volume harnessing water intake under scarcity regulated by AMF.

Several studies have illustrated the varied plant responses to drought owing to the specificity of AMF to plant roots [30,31,32,33,34,35,36,37,38,39,40,41,42,43]. Randomized metabolomic analysis in tomato roots colonized by three AM fungi of different genera revealed that some responses to drought and salt stress were commonly mediated by most of the AM fungi, whereas some were specifically associated with single isolates. Single AMF inoculation on comparison with combinatorial assessment of three AMF species in studying tomato tolerance in mitigating water limitation showed variation benefits. Two AM fungal inocula (Myc_Rhizo and MULTISTRAIN) revealed a consistent difference compared to mixed inoculums that showed root inoculation pattern variation [44]. Consequently, mixed inocula (e.g., MULTISTRAIN vs. Myc_Rhizo) both in control and drought plants depicted varied significance affirming species-specificity in affecting physiological traits [44]. *F. mosseae* and *R. intraradices* were assessed and proved for multitude of benefits concerning species specificity in plant-microbe symbiotic interactions both above and below ground levels [45]. This effect can be attributed to increasing in internodes and its ratio for plant growth promotion in *R. intraradices* and considerably no positive implications upon *F. mosseae* inoculation showing discrepancy [29]. The discrepancy with the research by Rivero et al. [43] can be acclimatized to a variety of cultivars impacting varied growth results. So, from these results, we can conclude that indigenous AMF isolated along the individual regions owing to environmental implications will have prominent results rather than introducing a new family of AMF than ad hoc families could yield considerable results [46,47]. AM symbiosis enables mycorrhiza-host plant-mediated drought tolerance based on nutritional availability showing that even under low nutrition, mycorrhizal colonization aids in host plant nutrition and survival [48] (Table 1).

*R. intraradices* colonization under varied drought stress intensities resulted in increased N and P levels in both onion and tomato plants, wherein the former host plant was able to survive due to less negative water potential. The latter host plant depicted improved P nutrition along with higher N content in both roots and shoots. The mode of action corresponds to an increase in N demand impeding the mobility of NO_3_ ions under water deficit altering nutritional status during mycorrhizal colonization [35]. Further, *R. intraradices* inoculation resulted in a 14% increase in shoot P concentration of wild type tomato plants under well-watered conditions and a 23% increase under drought stress [42]. Pi uptake, transfer and delivery have been largely investigated in AM roots, resulting in the characterization of a symbiotic inorganic phosphate (Pi) uptake pathway [49,50,51]. Eight *PHT1* genes regulate tomato roots uptake of Pi in tomato [52] and *PT* genes are synthesized inductively by mycorrhiza [53,54]. AMF-induced *LePT3*, *LePT4*, and *LePT5* PT genes were detected in water surplus conditions as evident from previous research indicating the differential regulation of PT genes under drought stress [53,54,55]. Under water stress, they found a different regulation of the considered *PT* genes. Particularly, it is worth noting the opposite trend for the two genes involved in the direct Pi uptake from soil (*LePT1* and *LePT2*), independently from the presence of the AM fungus. Among the AM-inducible *PT* genes, *LePT3* seems not involved in response to water deficit [45]. *LePT4* and *LePT5* transcript levels both increased under water deficit with highest values in *F. mosseae*, in agreement with the higher P content found in the leaves of these plants in respect to control ones. Overall results suggest a major role played by *LePT2* and *LePT4* in promoting tolerance to water deficit, particularly under a severe condition, although this is dependent on the fungal species.

Multi-omics and culture-omics have been utilized largely nowadays for deriving at better plant varieties in sustainable agriculture. Tomato root transcriptome analysis revealed that cytochrome P450 genes were mainly up-regulated in *R. intraradices* under drought stress [56]. Handa et al., [57] showed the importance of cytochrome P450 in the synthesis of sterols for membrane biogenesis during arbuscule formation, it could be inferred that their over expression reflects a change in fungal development under water deficit. Other most highly up-regulated fungal genes include a “conidiation protein 6” domain and domains involved in signaling transduction were significantly influenced by the WS treatment, being mainly up-regulated. Fungal conidiation can be induced by nutrient deprivation or mycelium desiccation, under drought stress. Moreover, they found three glutathione S-transferases (GSTs) were overexpressed in *R. intraradices* upon water stress. GSTs are recognized as a candidate in the cell protection from oxidative damage under drought stress both in tomato and fungi [56,58]. Such analysis for stress management will provide deeper insights for symbiotic management for multiple stress modalities.

### 4.2. Photosynthesis and Water Status

Tolerance elicited by AMF symbiosis in enhancing plants efficiency in fighting water insufficiency comprise increased water intake through Gs [39], efficient water use and prevention of reactive oxygen species production to reduce oxidative stress damages encompassing various antioxidant mechanisms, both enzymatically and non-enzymatically [59,60]. Affirmatively, a better performance of PS II was correlated to AM tomato plant interaction during water deficit and surplus [38]. Under field conditions also the symbiosis confirmed higher leaf relative water content (RWC) irrespective of various drought stress conditions in mycorrhizal inoculated tomato plants [35]. In a similar study, escalated photosynthetic activity, Gs levels in well-watered conditions and an increase in Pn and Gs values under stressed conditions provide clues for the efficiency of mycorrhizal infection in tomato plant growth upsurge. This was proved by the fact that recovery under fully watered conditions yielded neither Pn nor Gs [34]. The enhanced Gs probably caused an increase in intracellular CO_2_, which could also enhance the photosynthetic activity of the infected plants. These increases in the photosynthetic rate and leaf conductance were accompanied by the better uptake of water by the roots, as can be seen from the root hydraulic conductivity values. The results confirm that leaf water potential remains higher in mycorrhizal plants under drought conditions because of higher water uptake.

In a more recent study, *R. intraradices* colonization caused high Tr of mutant plants for abscisic acid (ABA) genes under well-watered conditions (*p* < 0.05) but not for *Tr* of wild type plants under drought stress. On the contrary, WUE of wild type plants under drought stress (*p* < 0.05) increased. Nevertheless, WUE under well-watered conditions did not show a considerable rise. Whereas Gs of non-inoculated tomato plants (*p* < 0.05) show a consequent increase when compared to the negative effect on colonized tomato plants. Drought significantly decreased the photosynthetic rate of non-inoculated plants (*p* < 0.01), but not for inoculated plants. Further, mycorrhizal inoculation depicted positive outcomes on the photosynthetic efficiency on two genotypes subjected to drought stress (*p* < 0.05) [42]. AM plants under drought stress substantially enhanced the Gs (increased by 62% with *S. deserticola* and by 200% with *S. constrictum*) in comparison to non-AM plants. Besides, leaf water potential and relative water content were higher in the presence of mycorrhiza, suggesting that AM plants could improve the water status under water deficit. Application of *S. constrictum* triggered a higher Fv/Fm in plants compared with non-AM and *S. deserticola* colonized plants under drought stress, which supports the results of Ruiz-Lozano et al. [38] that showed similar results under moderate and severe drought conditions [28]. AM plants inoculation illustrated efficient PS II and plant growth right from early inoculation than non-AM plants during drought stress [38]. Chitarra et al. [29] found that *R. intraradices* inoculation significantly increased the photosynthetic rate and water use efficiency. As a result of higher water status and improved nutritional status, mycorrhizal tomato plants have produced higher biomass under varying intensities of drought stress. The improved nutritional status and relative water content caused by mycorrhizal colonization would have alleviated drought impacts and promoted tomato fruit production under varying intensities of drought stress. Because mycorrhizal treatments consistently increased fruit yields under varying intensities of drought, WUE of AM plants were much higher than the control plants [29].

Also, the fungal external hyphae can penetrate pores that are beyond the root zone or inaccessible for the root systems, thus allowing colonized roots to access additional water reservoir in the soil [39]. Therefore, the differences found between mycorrhizal and non-mycorrhizal tomato plants were not due to osmotic adjustment, and the enhanced development of extraradical hyphae in soil containing mycorrhizal plants could be responsible for improving the root hydraulic conductivity and reducing drought stress in infected tomato plants [61]. Infection plays a pivotal role in increased water transport by hyphae directly than non-infected roots due to increased hydraulic conductivity and transpiration rate. Hydraulic conductivity transmits and sends the hydraulic message from the roots to the host plants as affirmed by high Gs levels [62].

### 4.3. Modification of Hormonal Balance

Furthermore, AM fungi modulated root hydraulic properties, regulated patterns of expression of aquaporin genes [63,64], induced changes in phytohormone ABA, JA [29,60], and altered proline content in the host plants [29]. AM symbiosis is critically mediated by ABA for successful regulation [65], on the other hand, AMF also regulates the ABA signaling pathway [66]. However, few evidences were available as to the interplay of AM symbiosis with the functional genes in ABA signal transduction under drought stress. In Xu et al.’s [42] study the ABA-deficient tomato mutant *not*, together with its wild-type was adopted to reveal the influence of AM symbiosis on 14-3-3 genes in response to drought stress. Expression variant profiling revealed high expression of 14-3-3 genes *TFT2*, *TFT3* and *TFT5*, *TFT7*, *TFT9* and *TFT10* in wild type plants and *not* plants, respectively. These genes further mediate the stomatal behavior, subsequently improving plant WUE and drought tolerance.

Neither drought nor mycorrhizal inoculation influenced the ABA concentrations in *not* leaves [42]. Furthermore, AM-induced alterations such as hormonal changes, improvement of gas exchange, regulation of osmotic adjustment, and mediated aquaporin genes in host plant as well as in fungal symbiont can also contribute to the host water status [25,29,64]. The expression of *LeNCED1* was not regulated by drought or by ABA in tomato plants under drought stress [36,57,67]. However, the expression pattern of the ABA-responsive gene *Le4* perfectly matched with that of ABA levels, indicating an efficient activation of the ABA signaling pathway under drought. In addition to its role as a “stress phytohormone”, ABA is also important for symbiosis establishment and functioning [65,68]. Therefore, the increased ABA levels in stressed plants would serve not only to promote tolerance against stresses in non-AM and AM plants but also to enhance and maintain the symbiosis in mycorrhizal plants. Hormonal results, together with those of other physiological parameters, support that AM symbiosis improves plant fitness under water-related stress conditions. Although ABA has not been detected in Rivero et al. [43] study, instead they found higher concentrations of ABA-GE in mycorrhizal roots, a reservoir for the rapid production of active ABA. Same results were found in Chitarra et al. [29], under severe WS conditions (21.3 MPa), tomato AMF inoculated plants showed significantly lower levels of ABA in roots compared with control plants. The expression of the ABA biosynthetic gene *LeNCED1* was low and constant in the root of NS plants, without significant changes due to the presence of AM symbionts. Under drought stress, a strong increase in the transcription of this gene was observed in control plants, while in AM-colonized plants under drought stress, *LeNCED1* was only up-regulated in *F. mosseae* plants, whereas it did not undergo significant expression changes in roots from the *R. intraradices* inoculated plants [29]. At the same time, the gene was also vague in showing less regulation because AM colonization could vary in yielding significant results based on the experimental setup and system variations [38].

ABA biosynthetic gene *SlNCED* was downregulated under *S. constrictum* colonization under drought conditions. Nevertheless, the levels were normal and optimized without stress [58]. Conversely, significantly higher expression levels of JA-biosynthetic gene *SlLOXD* in roots colonized by *S. constrictum* were observed under non-stress and drought conditions. There are ample reports that confirm ABA in structuring and maintaining AMF symbiosis [65,68] at the same time, JA plays a pivotal role and is involved in organizing and developing AM symbiosis [69,70]. Under drought stress and high salinity conditions, water loss is prevented by closure of stomata by ABA production [71], therefore down-regulated *SlNCED* in mycorrhizal plants relates to higher stomatal opening and conductance and elevated water status of the host plants. The above reasons can be enlisted for stress tolerance in AMF inoculations. Increased production of strigolactones has been reported in lettuce and tomato plants under drought in the presence of *R. irregularis*, indicating that AMF symbiosis induces striglolactones biosynthesis [38].

Expression profiling for *SlCCD7* and *SlCCD8* genes responsible for striglolactones biosynthesis revealed upregulation of *SlCCD7* under drought stress without *SlCCD8* gene alterations. Strigolactones are increased upon drought stress to promote AM symbiosis in the regulation of host plant development. But further research is needed to confirm the intrinsic correlation between striglolactones biosynthesis and AM symbiosis under drought conditions. Striglolactones—ABA crosstalk in plant retorting to water stress show the mechanistic background for colonization criteria for AM symbiosis. *F. mosseae* inoculated plants show high proline values under inoculation in drought plants rather than control plants. AM symbiosis could not be relied on drought conditions, as varied expressions of proline content in treated plants than in control plants remains clueless [37,72,73,74]. Our results, showing a higher content of proline in AM inoculated plants during drought concerning control plants, could suggest a better resistance of colonized plants to drought.

Physiological mechanisms in averting drought stress are regulated by the expression of genes encoding aquaporin proteins (AQPs) that controls water movement in plants [75]. Furthermore, AM symbiosis can have potential implications in regulation of the expression profiles of AQP genes to enhance root hydraulic conductivity as well as the plant water status and tolerance under water deficiency. Currently, five major subfamilies of AQPs have been characterized based on sequence similarity [76]. Drought affected the transcriptional pattern of these AQP genes differently: *LeNIP3;1* was over-expressed in AMF-inoculated plants, particularly in those inoculated with *F. mosseae*; conversely, *LePIP1;1* and *LeTIP2;3* were down-regulated in both AMF inoculated and control plants [29]. AM fungal AQPs can also have a role in drought tolerance during AM symbiosis, the expression profiling for two *R. intraradices* AQP genes (*RiAQPF1* and *RiAQPF2*) indicated up-regulation of *RiAQPF2* gene under drought suggesting putative roles in drought stress response [29]. The simultaneous induction of both fungal and plant AQP genes also affirm that the two symbionts strictly cooperate to regulate the mycorrhizal drought stress response resulting in enhanced physiological performance [29].

## 5. Salt Stress

Salinization of soil is a serious agricultural and eco-environmental problem and is increasing steadily in many parts of the world, in arid and semi-arid areas. Salinity inhibits plant growth by lowering soil water potential and increasing the potentially toxic ions (Na^+^ and Cl^−^), which in turn lead to water deficit and nutritional imbalance. Increasing Na^+^ concentrations disturb the nutrient balance, osmotic regulation, and cause ion toxicity [77,78]. Salinity stress significantly reduced the root, stem, and leaf dry matter and leaf area compared with the control treatment due to direct effects of ion toxicity or indirect effects in osmotic imbalance due to increase in saline ions between soil and plant. In the areas with an optimal climate for tomato cultivation, salinity is a serious constraint [79]. Salinity irrevocably affects plant length, dry weight, and growth of tomato plants. Salinity also reduced the fresh and dry shoot and root weight of tomato [80,81]. The reduction of dry weights due to increased salinity may be a result of a combination of osmotic and specific ion effects of Cl and Na [82]. Salinity increased the absorption of Na^+^, whereas it decreased N, P, K, and Mg uptake, and K^+^/Na^+^ ratio. AMF has been extensively identified as a sensible fungus in salinity adversities on host plants under salt stress [83] by negatively regulating salinity impacts by mycorrhizal symbiosis [84]. Nevertheless, many literatures show that AMF can enhance salt tolerance of many plants and some physiological changes occurred in AM symbiosis under salt stress [80,82,85]. In recent years, studies indicated that AMF can increase plant growth and uptake of nutrients, decrease yield losses of tomato under saline conditions and improve the salt tolerance of tomato [80,82,86,87].

### 5.1. Tomato Growth and Nutrient Uptake

Previously, some studies have indicated that AMF can enhance the plant growth and uptake of nutrients, decrease yield losses of tomato under saline conditions, and improve salt tolerance of tomato [86,87,88]. Colonization with *F. mosseae* significantly improved dry matter and leaf area in the salt-stressed tomato plants. This effect of AM on the dry matter was more pronounced in aerial biomass than in root biomass which may be because arbuscular mycorrhizal colonization was proportionally greater in roots than in shoots concerning the distribution of carbohydrates. Under salinity, *R. intraradices*-mediated growth stimulation was higher in more salt-tolerant tomato cultivar, Piazar than sensitive cultivar, Behta. High salinity reduced the dry matter production of plants in both cultivars [89]. Interestingly, soil salinity accounts for the AMF inoculated tomato plants to show increased dry matter (0.098 g plant^−1^) content than non-inoculated seedlings (0.082 g plant^−1^) [90]. Beneficial effects of AM fungi have been appropriated not to root biomass rather on above-ground biomass [83]. P nutrition was partially linked to better growth under salinity due to mycorrhizal inoculation in tomato plants [91]. AMF-enriched plants grown in saline soils (1.5 mS cm^−1^ to 6.0 mS cm^−1^) showed amplified fruit yield, quality, mineral composition, and antioxidant status. As for fruit mineral composition [88], the application of AMF-based formulates resulted in higher content of K, Ca, Mg, P, S, and NO_3_ compared to the control, whereas Cl was not significantly affected by the beneficial microorganisms.

AMF inoculation showed low Na levels in fruits apart from non-inoculated counterparts due to concomitant accretion of K, Ca, and Mg. N, P, Mg, Ca, Mn, and Fe uptake was subsequently high in tomato seedlings inoculated with AMF under salt stress than non-inoculated ones. In other research [15], plants inoculated with *R. intraradices* produced fruits with a higher content of K, Ca, P, and Zn compared to control plants. Moreover, mycorrhization increased P, K, and Ca contents more in salt-tolerant Piazar than salt-sensitive Behta [82]. Different mycorrhizal responsiveness of cultivars could be the result of plant- and/or AMF-related mechanisms. The enhancement of plant P and K uptake by AMF have been reported and was considered one of the main reasons for the amelioration of growth in salt-affected plants colonized by AMF [92]. Earlier studies found that the improved growth of mycorrhizal plants in saline conditions is highly related to the mycorrhizal-mediated enhancement of host plant P nutrition [93]. AMF in salt-stress is responsible for increased P uptake due to integral vacuolar membranes and compartmentalization Na^+^ ions regardless of integral P in host plants [94]. Recently, Ebrahim et al. [85] found that the AMF inoculation (*R. fasciculatus*) increased the accumulation of N, P, K, and Mg, it reduced the Na^+^ concentration. Moreover, AMF can increase the accumulation of osmotic regulators such as soluble sugar [95], proline, betaine, and polyamines [96,97] in plants under salt stress. Kong et al. [98] confirmed that the tomato plants and mixed inoculation of AMF (*R. clarum* and *R. intraradices*) formed a symbiotic relationship that significantly improved the growth of plants and increased the concentration of vitamin C, soluble sugar and lycopene in the tomato fruit (Table 2). Compared with the control, the single fruit weight and the yield per plant in the AMF treatment were significantly increased. AMF promoted tomato plants absorption of N and reduced the absorption of Na^+^. Under NaCl salinity, *R. etunicatum* than other AMF counterparts *F. mosseae* and *R. irregular* enhanced plant growth promotion mainly the root growth [41].

### 5.2. K^+^/Na^+^ Ratio

Because reduced growth under salinity is partially caused by ion imbalances and/or non-availability of nutrient ions due to their competition with major ions (Na^+^ and Cl^−^) in the soil [99], the sustained growth of AMF+ plants under salinity is partially based on improved uptake of nutrients and maintaining favorable ionic ratios [100]. AMF inoculation can maintain the K^+^/Na^+^ balance under salt stress. AMF restricted the transport of Na from roots to shoots in plants, which increased K^+^/Na^+^, Ca^2+^/Na^+^, and Mg^2+^/Na^+^ in leaves and stems, in addition to protecting the photosynthetic organs from damage. Certain ion ratios, such as K/Na, are accepted indicators for the evaluation of salinity tolerance in tomato cultivars. Hajiboland et al. [82] reaffirmed that AMF inoculation depicted high content of K/Na and Ca/Na levels equally in roots and shoots than non-inoculated plants. Mycorrhizal *F. mosseae* plants had a higher concentration of K at both salinity levels [89]. Na concentration was lower in mycorrhizal than non-mycorrhizal plants regardless of the salinity level. Growth improvement was observed in AMF-inoculated tomato plants under salinity conditions and was mainly associated with ionic factors such as higher K concentration and K^+^/Na^+^ ratio [101]. Indeed, although K concentration and the K^+^/Na^+^ ratio in leaves were positively correlated with the growth parameters, those correlations were especially apparent in the AMF-inoculated plants. The concentration of Na^+^ in roots was reduced in mycorrhizal treatments, while the accumulation of Na^+^ in leaves was significantly lower in plants colonized by *R. etunicatum* [43]. Notably, *R. etunicatum* was the only salt-adapted AMF compared to *F. mosseae*, *R. irregulare*. Higher K accumulation by mycorrhizal plants in saline soil could be beneficial by maintaining a high K/Na ratio and by influencing the ionic balance of the cytoplasm or Na efflux from plants. The higher K accumulation in AM plants under salt stress conditions resulted in maintaining a high K/Na ratio, preventing the disruption of metabolic processes and the inhibition of protein synthesis [82,102]. AQP gene (*GintAQP1*) in *R. intraradices* expressed upon homeostasis relying on two Na^+^/H^+^ antiporters in salinity stressed tomato plants depict insignificant expression levels of *LeNHX1* and *LeNHX2* genes under AMF symbiosis [103].

### 5.3. Photosynthesis and Water Status

AMF also increased the chlorophyll concentration, Pn, Gs, and Tr of plants. AMF infection effectively improves plant photosynthetic capacity [104,105], and salt stress-induced stomatal closure [106]. Colonization with AMF enhanced CO_2_ assimilation rate of salt-affected tomato plants. *R. intraradices*-inoculated tomato plants had greater transpiration and stomatal conductance. AMF inoculation consequently lowered WUE than non-inoculated counterparts. *R. fasciculatus* role in enhancing chlorophyll concentration can be ascribed to increase in N and Mg concentrations and reduction in absorption of sodium in tomato leaves. Light-harvesting protein complexes variability patterns can be corroborated to salinity stress tolerance and adaptations based on the rise in Chl a/b ratio [85]. AMF also enable processing tomato plants to accumulate more proline and to reduce membrane peroxidation damage [98]. Oxylipins such as the phytohormone JA and derivatives reduced the negative effect of drought and salinity stress on plant fitness when exogenously applied [107]. The AM colonization resulted in the accumulation of JA; the higher the JA concentration in the roots colonized by *R. etunicatum*, the more efficient the bio-protector against salt stress. ABA glucosyl ester (ABA-GE), b-ionone, and blumenol C glucoside belonging to the carotenoid pathway are other metabolites quantified in the *R. etunicatum*-colonized AM roots apart from oxylipins [43].

### 5.4. Antioxidant Enzymes

Reactive oxygen species (ROS) such as superoxide radical (O_2_^−^), hydrogen peroxide (H_2_O_2_), hydroxyl radical (OH), and singlet oxygen (O_2_^−^) are generated in plants during the salt stress. The oxidative stress results in oxidative stress-mediated damage to the vital cell components like proteins, nucleic acids, and lipids. This led to the change in selective permeability of bio-membranes and thereby membrane leakage and change in the activity of enzymes bound to the membrane occurred. The induction of ROS-scavenging enzymes, such as superoxide dismutase (SOD), peroxidase (POD), ascorbate peroxidase (APX), and catalase (CAT) is the most common mechanism for detoxifying ROS synthesized during stress responses. AMF can also reduce the level of ROS by increasing the activities of antioxidant enzymes, and other enzymes under stressful conditions. The activity of antioxidant enzymes (SOD, CAT, POD, and APX) increased in *F. mosseae*-inoculated tomato plants under salinity (except SOD and CAT activity at 100 mM NaCl). However, this higher activity did not provide enough protection against ROS, as judged by simultaneous enhancement of MDA [89]. Under saline conditions, ionic balance involved in photochemical reactions and gaseous exchange is regulated by enhanced proline concentration, lowered lipid peroxidation, and increased SOD activity [82].

Inoculation with AMF increased the activity of antioxidant enzymes and protein contents of salt-affected plants. AMF colonization was accompanied by an enhancement of the activity of APX, CAT, and SOD in both salt-affected and control tomato plants. Besides, inoculation with AMF caused increase in the proline concentrations and reduction of hydrogen peroxide and MDA [82]. The role of ROS-scavenging enzymes in AM tomato under salt stress showed the positive effects of AMF on tomato growth [80], which corroborates to a similar study wherein lower leakage value was found for macromolecules and MDA content in AM plants under salt or saltless condition. The lower leakage values for macromolecules and MDA content illustrates lower cell membrane damage or higher salt tolerance [87]. AM inoculation-enhanced tomato salt tolerance assessment shows that cell membrane damage was lower in AM symbiosis compared to non-AM plants and SOD, APX, and POD activity was contributed to protecting the plant from salinity injury, the induced SOD, APX, and POD activity in AM symbiosis may be an important mechanism to improve salt resistance of AM plants [80].

## 6. Temperature Stress

The optimal temperature for tomato cultivation ranges from 20 to 30 °C. Stress due to heat will have profound implications on growth parameters and result in major threats in a plethora of plant parameters inducted by adversities in physiological, biochemical, morphological, anatomical, and genetic responses in plants [108], eventually depressing yield and the quality of crops. Tomato usually prefers thermophilic environment stating that the plant is susceptible to various temperature conditions. Physiology and metabolic regulations are the key components in growth and improvement in plants which are impeded by a reduction in temperature. Low-temperature conditions will affect the normalized distribution, water potentials, and photosynthetic ability. The entire scenario can be associated with stomatal conductance irregularities by altercations in hydraulic conductance [109,110]. Also, low temperature decreases the capacity and efficiency of photosynthesis through the change in pigment composition, a decline in chlorophyll fluorescence, and impaired chloroplastic development [105]. When the plant is subjected to low-temperature stress, the cell membrane is first affected with increased membrane permeability. At the same time, a variety of ROS, such as O_2_^−^, OH, and H_2_O_2_, are induced, causing a loss in the balance between production and scavenging in the cell or organism, which causes membrane lipid peroxidation [111].

Most of the cultivated processing tomato genotypes are sensitive to low temperatures (0–12 °C) in all growth stages [112]. The negative effects of chilling are more remarkable during germination and at the seedling stage [113]. Chilling damage could limit processing tomato growth and production in an open field. Inoculation with *F. mossae* was the most effective treatment in reducing electrolyte leakage while increasing the efficacy of PSII and regrowth capacity of seedlings exposed to severe chilling stress (1 °C) [114]. *F. mosseae* has been proved responsible for escalations in redox compounds enrooting stability of tomato roots in optimal temperature conditions [115]. The phenomenon underlying is being reinstated of the chilling stress in seedlings to overcome the temperature complications. Similarly, they also proved that AMF-inoculated tomato seedlings exhibited significantly higher fresh weight and dry weight than non-AMF control plants under both control (25 °C/15 °C) and low temperature (8 °C/4 °C) treatments. Under chilling stress, AMF inoculation significantly reduced the level of MDA, H_2_O_2_, and O_2_^−^ than in the non-AMF control because of calcium precipitation in tomato roots apoplast and vacuole. Furthermore, AMF inoculation induced activities of antioxidant enzymes and transcripts of related genes under chilling stress.

AM fungi have largely been studied to tackle low temperature in addition to boosting tolerance to stress mechanisms both biotic and abiotic together with plant growth promotion [110,116]. *F. mosseae* grow better than non-mycorrhizal plants under low temperature [117]. A low chlorophyll concentration in non-mycorrhizal and low temperature-stressed tomato plant leaves indicated a reduced synthesis rate of chlorophyll and an increase in chlorophyll breakdown. Mycorrhiza and low temperature were reported to modify the protein content in tomato, whereas an increase in soluble proteins was related to low-temperature tolerance [117]. *F. mosseae* could enhance chlorophyll concentration of tomato leaves at low temperature, which was in agreement with the results of wheat and maize under cold stress by Paradis et al. [118] and Zhu et al. [110]. Proline agglomeration can cause deleterious effects of temperature stress; mycorrhizal plants evidenced lower accumulation than non-mycorrhizal plants. MDA content in mycorrhizal plants remained lower than that in non-mycorrhizal plants indicating that leaf proline can have indirect consequences in addressing osmotic imbalance and lipid peroxidation mechanism alleviation by AM fungi will have protective effects than lowering proline accumulation in the tomato leaves [117]. They also reported the activities of SOD, POD, and APX in AM tomato plants were higher than the non-AM plants under low-temperature stress. An increase in MDA level and H_2_O_2_ accumulation in the leaves of stressed plants, although the levels of MDA and H_2_O_2_ were lower in AM plants than in non-AM plants according to the different stresses [28]. Similar results were obtained from *R. versiforme* inoculation during drought [119] and *R. intraradices* inoculation under salt stress salinity [82], show similar consistent results for leaves and roots of citrus seedlings. All these results summarily prove AM colonization is responsible for enhancing the survival rates under extreme temperature by lipid peroxidation and amelioration by ROS deleterious effects. Recently, Haddidi et al. [120] showed the beneficial role of AMF symbiosis in the alleviation of ROS accumulation caused by combined drought and heat, and combined drought and heat shock stress. They revealed that the accumulation of H_2_O_2_ and lipid peroxidation was much higher in leaves than in roots. However, inoculation with different AMF strains, and especially, *F. moseae*, could enhance tomato plants’ tolerance by lowering H_2_O_2_ and MDA content, and changed the activities of antioxidant enzymes (Table 3).

## 7. Heavy Metal Stress

Heavy metals (HMs) such as arsenic (As), lead (Pb), mercury (Hg), cadmium (Cd), chromium (Cr), nickel (Ni) and copper (Cu) are toxic based on their toxic effects on human beings [121]. Not all metals are harmful to possess severe and devastating health effects, rather micronutrients potential pose some of the metals to have immense biological properties for the welfare of human beings and agricultural potentialities. Whereas non-essential metals have irrevocable consequences on the environment and ecosystem thereby soil health and fertility are affected badly. The food chain and food web are massively affected posing severe threats to human health by long-term effects on plants and food products. Biological methods of plants-microbe’s interactions can be used for the bioremediation of contaminated areas with heavy metals. AM fungi colonization and benefits do not depend on soil pollution even in the extreme terrestrial environment [122]. AMF can be adapted to various environmental conditions even in metal-contaminated sites [123] (Table 4). AMF-colonizing plant roots will alter the morphological parameters of plant roots and nutritional status of the soil [124,125], mycelial culmination and adsorption of heavy metals [126], and secondary metabolites-mediated mitigation of toxic HMs concentration and metal resistance, finally aiding efficient growth through the accumulation of key molecules like glomalin, organic acids, and auxin [127,128]. Tomato plants subjected to Cd stress showed reduced growth in terms of length and weight [129]. Cd alters growth, triggers leaf necrosis and impedes cell division, elongation, and reduced growth attributes in tomato [130]. Chlorophyll content in AMF-inoculated tomato plants and subsequent recovery of Cd-stressed plants may have effects due to the effect of AMF on magnesium uptake. Proline and sugar accumulation help plants to maintain cellular water potential well below that of the soil solution [129]. Cd stress has positive attributions for proline accumulation in tomato [131] which emancipates a positive correlation between AMF symbiosis and proline accumulation under severe stress. The role of proline together with AM inoculation suggests increased levels of scavenging enzymes like SOD, CAT, POD, GR, and APX under Cd stress through antioxidant quenching of ROS [129]. *F. mosseae* when inoculated in Cd contaminated tomato plants considerably enhanced biomass of shoots and roots. These effects were substantiated to Cd sequestration in non-vital mycorrhizal roots and Cd immobilization due to microbial adsorption, accumulation, and/or chelation by rhizosphere beneficial effects [132]. Further, *F. mosseae* drastically reduced toxic effectuations upon cadmium accumulation. The results depict that pre-transplant inoculation incited plant dry matter accumulation than post-transplant inoculation despite cadmium contamination richness in the soil [133]. On the contrary, AM inoculation did not elicit considerable alleviation of Cd contamination due to intra-radical compartmentalization in root cell vacuoles which results in translocation of Cd in aerial roots resulting in normal growth and productivity. Furthermore, tomato grafting with Maxifort rootstock minimized Cd-induced oxidative injury hydrogen peroxide, lipid peroxidation, and electrolyte leakage in tomato leaves and thereby promoting the growth performance of tomato plants. These results are attributed to alterations in plant nutritional status, photosynthetic pigments, the photochemical activity of PSII, increase the capacity of antioxidant enzymes (CAT, APX), proline and metabolites linked to oxidative stress clearly related to Cd tolerance (i.e., phytochelatin, fructans, and inulins) [134]. *F. mosseae* BEG167 under field conditions were evaluated for pot experiment analysis for tomato plants contaminated with five As levels (0, 25, 50, 75, and 150 mg kg^−1^) and found that mycorrhizal colonization increased plant biomass at application rates of 25, 50, and 75 mg kg^−1^. Mycorrhizal colonization under the rate of 75 and 150 mg kg^−1^ concentration levels were escalated in shoots, whereas decreased in 50 mg kg^−1^. Total P was supplemented at the levels of 25, 50, and 75 mg kg^−1^, and increased supply was administered to mycorrhizal colonized roots which demonstrated higher shoot and root P/As ratio than the non-mycorrhizal counterparts. pH values of the soils were high in uninoculated controls indicating that AM fungi can have enormous applications in phytostabilization of HMs-contaminated soils and phytoextraction of the contaminated metal pollutants for soil enrichment and enhancement for growth optimization [135].

Mycorrhizal colonization was subjugated for evaluation under arsenic supplemented soils for tomato seedlings upsurge and enhanced shoot height and root length was found in inoculated soils than in non-inoculated control soils. Vegetative growth was linked toward the positive outcomes and nutrient availability and uptake. High chlorophyll content with a decrease in arsenic levels in mycorrhizal colonized plants can also be connected to translocation of metal ions from soil to tomato plants. This criterion can also be rationalized for AMF effect on P uptake [136]. Cu pose as yet another useful micronutrient that is indispensable for plant growth and productivity. *R. intraradices* and *R. etunicatum* inoculation were experimented against challenged soils with increased Cu and illustrated that dry weight of roots and shoots increased quantitatively and qualitatively [137] (Table 4).

These results can be related to chlorophyll content and Cu translocation pattern as seen in arsenic supplementation. Further, AM fungus and host plant interaction show shooting up of protein synthesis in elucidating oxidative enzyme levels under metal stress. The results of the findings escalate the idea that involvement of stress proteins like phytochelatins and metallothioneines will have possible roles in mechanistic protection [138,139]. At the same time, copper has its ill-effects in the metabolic hindrance of cellular activities and protein synthesis machinery which ultimately affects the total protein content in non-mycorrhizal inoculated tomato plants providing a clue to search for deeper insights for the assessment and confirmation. With this notion, it was evident that soil pollution with toxic heavy metals showed a considerable rise in enzymatic levels involved in antioxidant properties viz, APX and GUPX [139]. Thus, it is evident that the review addresses the physiology, inoculation efficiency, phenomenal interactions mediating the plant-microbe association for beneficial aspects of the involvement of AM fungi in combating a variety of abiotic stress modes and effective research perspectives in mitigating the stress and abatement strategies like sequestration, oxidative stress quenching, stress proteins involvement, and metal chelating proteins along with other essential modalities. Thus, the review compiles rationally the approaches to tackle the abiotic stress arresting by AM fungi and other molecular plant strategies in the apprehension of stress and establishment of sustainable plant growth not only for tomato but also other horticultural crop plants.

## 8. Concluding Remarks and Future Prospects

The stress biology of tomato has been vehemently researched worldwide to substantiate the increasing population need for copious food. This comprehensive analysis demonstrated the molecular mechanisms underlying abiotic stress physiology and combating strategies abated by AMF. However, photosynthetic efficiency (Pn, Gs, Tr, Fv/Fm), root morphology, hydraulic conductivity, elicitors’ modality in arresting stress mechanisms remains to be explored still. The practice of genetic engineering and microbial biotechnology resulted in more investigation on development of plant tolerance possible, even when conventional breeding reached its limits. Most recently, it was found that clustered regularly interspaced short palindromic repeats (CRISPR)/CRISPR-associated protein 9 (Cas9) systems are significant ameliorators in the regime of biotic and abiotic stress and plant-microbe interactions. Genome editing provides target sequence tailored modifications through agricultural trait improvement. Moreover, transcriptome analysis for stress management will provide deeper insights for symbiotic management for multiple stress modalities. In the present scenario, this review will allow agricultural scientists to explore the potentials of AMF, and further, it will act as a launchpad for effective sustainable agricultural practices.

## Figures and Tables

**Table 1:** Application of arbuscular mycorrhizal fungi on drought stress alleviation.

Mycorrhizal Species	Effect	Reference
*Septoglomus deserticola* and *Septoglomus constrictum*	Increased root and shoot dry weight. Increased stomatal conductance, relative water content and antioxidant enzyme activities	[28]
*Funneliformis mosseae* and *Rhizophagus intraradices*	Increased photosynthetic rate and water use efficiency, reduction in ABA, higher proline accumulation,	[29]
*Glomus clarum*	Increased aerial biomass, photosynthetic activity and stomatal conductance	[34]
*Rhizophagus intraradices*	Increased shoot biomass, number of flowers, fruits, N and P uptake.	[35]
*Rhizophagus intraradices*	Increased shoot dry weight, stomatal conductance, photosystem II efficiency, and abscisic acid	[38]
*Rhizophagus intraradices*	Lower transpiration rate and increased water use efficiency, increased shoot biomass and P concentration	[42]
*Funneliformis mosseae, Rhizoglomus irregulare* and *Rhizophagus etunicatum*	Increased shoot and root dry weight.	[43]
*Funneliformis mosseae* and *Rhizophagus intraradices*	*Rhizophagus intraradices* efficient in water use efficiency, *F. mosseae* in volatile organic compounds	[45]

**Table 2:** Application of arbuscular mycorrhizal fungi on salt stress alleviation.

Level of Salinity	Mycorrhizal Species	Effect	Reference
NaCl solution (0, 0.5 and 1%) 4.2 and 7.1 dS m^−1^	*Funneliformis mosseae*	Reduced MDA content and increased antioxidant enzymes SOD, POD, APX and CAT	[80]
without salt (EC = 0.63 dS m^−1^), with low (EC = 5 dS m^−1^), or high (EC = 10 dS m^−1^) salinity.	*Rhizophagus intraradices*	Increased growth, stomatal conductance, photosynthetic activity, proline and ROS enzymes	[82]
1.4 (control), 4.9 (medium) and 7.1 dS m^−1^ (high salt stress)	*Funneliformis mosseae*	Shoot dry matter, yield and leaf area were higher, the contents of P, K, Zn, Cu, and Fe were higher	[87]
1.5, 3.0, 4.5, 6.0 mS cm^−1^ EC	*Rhizophagus etunicatum*, *Funneliformis mosseae*, *Glomus aggregatum*, *Rhizophagus intraradices*	Increased yield and size of fruits	[88]
0, 50 and 100 mM NaCl.	*Funneliformis mosseae*	Increased growth, leaf area, cholorphyll, fruit fresh weight and yield. Increased P and K uptake. Increased SOD, CAT, and POD reduced MDA content	[89]
0, 50, and 100 mM, NaCl	Mixtures of *Glomus* sp.	Increased root biomass, P, N, Ca uptake	[90]
EC of 4.56 dS m^−1^	*Glomus clarum* and *Rhizophagus intraradices*	Increased soluble sugar, proline accumulation and vitamin C. Increased the chlorophyll concentration, Pn, Gs and Tr of plants.	[98]

**Table 3:** Application of arbuscular mycorrhizal fungi on temperature stress alleviation.

Type of Temperature	Mycorrhizal Species	Effect	Reference
42 °C	*Septoglomus deserticola* and *Septoglomus constrictum*	Decreasing the lipid peroxidation, hydrogen peroxide level and improving leaf and root antioxidant enzyme activities	[28]
1 °C	*Funneliformis mosseae*	Reduced the cell membrane injuries in term of electrolytic leakage and efficiency of photosystem II	[114]
8 °C/4 °C	*Funneliformis mosseae*	induced activities of antioxidant enzymes, significantly reduced the level of malondialdehyde (MDA), H_2_O_2_, and O_2_^−^ along with increased calcium precipitates in the apoplast and vacuole of root cells	[115]
8 °C	*Funneliformis mosseae*	Decreased MDA content in leaves. The contents of photosynthetic pigments, sugars and soluble protein in leaves were higher, but leaf proline content was lower and increased the activities of antioxidant enzymes	[117]
45 °C	*Rhizophagus irregularis, Funneliformis mosseae,* and *Funneliformis coronatum*	A decrease in hydrogen peroxide and malondialdehyde content and increased antioxidant enzyme activities	[120]

**Table 4:** Application of arbuscular mycorrhizal fungi on heavy metal stress alleviation.

Level of Heavy Metals	Mycorrhizal Species	Effect	Reference
Cd concentrations (50 and 100 mg kg^−1^)	*Funneliformis mosseae*	Increased dry weights, reduction in transcolation of Cd from root to shoot	[66]
Cd (50 μM CdCl_2_)	*Funneliformis mosseae Rhizophagus intraradices* and *Rhizophagus etunicatum*	Increased antioxidant enzymes, Proline and phenol content	[129]
Cd (30 and 60 mg of CdSO_4_)	*Funneliformis mosseae*	Increased Cd absorption and dry weights	[133]
0 and 25 μM Cd	*Rhizophagus irregularis*	Induced photosynthetic pigments, photosystem II efficiency, antioxidant enzyme and proline accumulation and reduced lipid peroxidation products. Increased nutritional status such as P, K, Ca, Fe, Mn, and Zn	[134]
As levels (0, 25, 50, 75 and 150 mg kg^−1^)	*Funneliformis mosseae*	Increased biomass and P uptake, higher shoot and root P/As ratio	[135]
Cu solution (0, 1.5, 3.5, 5.5, 7.5 mM CuSO_4_)	*Rhizophagus intraradices* and *Rhizophagus etunicatum*	Increased biomass, sugar, proline, and antioxidant enzymes	[137]
